# Pathogenic PPP2R5D variants disrupt neuronal development and neurite outgrowth in patient-derived neurons that are reversed by allele-specific knockdown

**DOI:** 10.1016/j.xhgg.2025.100450

**Published:** 2025-05-08

**Authors:** Randee E. Young, Michael V. Zuccaro, Charles A. LeDuc, Noelle D. Germain, Tae Hyun Kim, Patrick Sarmiere, Wendy K. Chung

**Affiliations:** 1 Department of Pediatrics, Boston Children’s Hospital, Harvard Medical School, Boston, MA 02115, USA; 2 Department of Pediatrics, Columbia University Medical Center, New York, NY 10032, USA; 3 Ovid Therapeutics, Inc., New York, NY 10001, USA

**Keywords:** neurodevelopmental disorders, PPP2R5D, Houge-Janssens syndrome, induced pluripotent stem cells, iPSCs, patient-derived neurons, allele-specific knockdown, antisense oligonucleotides, neurite outgrowth, RNA-seq

## Abstract

A significant barrier to the treatment of neurodevelopmental disorders (NDDs) is a limited understanding of disease mechanisms. Heterozygous missense variants in *PPP2R5D* cause Houge-Janssens syndrome 1, a rare NDD characterized by macrocephaly, developmental delay, intellectual disability, seizures, autism spectrum disorder, and early-onset Parkinson disease. This study investigated the impact of pathogenic *PPP2R5D* variants on neuronal development and evaluated allele-specific knockdown as a potential therapeutic strategy. Induced pluripotent stem cells derived from individuals carrying the E198K and E420K variants, along with CRISPR-corrected isogenic controls, were differentiated into neural progenitors and cortical glutamatergic neurons. Patient-derived neural progenitors were hyper-proliferative, and glutamatergic neurons differentiated from these cells exhibited increased neurite outgrowth. Notably, neuronal overgrowth phenotypes were not observed in neurons lacking *PPP2R5D*, suggesting the disorder does not result from loss of function. RNA sequencing (RNA-seq) of glutamatergic neurons derived from patient lines compared to their isogenic controls revealed disruptions in pathways critical for neuronal development, synaptic signaling, and axon guidance. To target pathogenic transcripts, antisense oligonucleotides (ASOs) were designed to selectively knock down the E198K allele, the most common disease-causing missense variant. The most effective ASOs reversed neurite outgrowth defects in patient-derived neurons. These findings uncover molecular mechanisms underlying PPP2R5D-related NDDs and support allele-specific knockdown as a potential therapeutic approach.

## Teaser

This work defines the cellular and molecular impact of PPP2R5D mutations in a rare neurodevelopmental disorder and shows that allele-specific antisense oligonucleotides can correct neuronal abnormalities, paving the way for precision therapeutics.

## Introduction

The protein phosphatase 2A (PP2A) holoenzyme is one of the major serine threonine (Ser/Thr) phosphatases and regulates many cellular processes and signaling networks, playing an important role during development.^1^ PP2A is a heterotrimer composed of scaffolding, regulatory, and catalytic subunits. The regulatory subunits of the PP2A holoenzyme are encoded by 15 genes, producing several combinations of Ser/Thr dephosphorylation holoenzymes. *PPP2R5D* (also known as B56δ; GenBank: NM_006245) is one of the PP2A holoenzyme regulatory subunits and encodes a 602-amino-acid protein.

**Figure 1:**
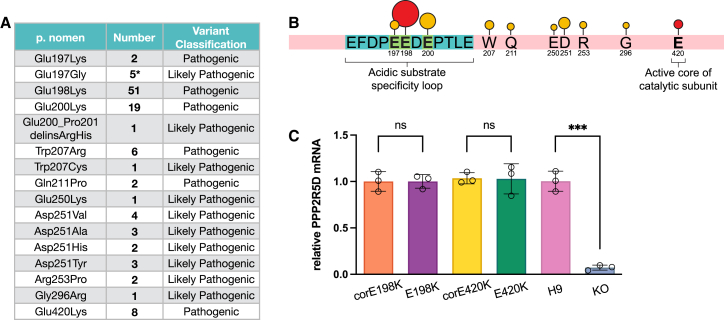
Overview of pathogenic *PPP2R5D* variants (A) List of pathogenic variants observed in individuals with PPP2R5D*-*related NDD. ∗ indicates inherited variant seen in one family. (B) Lollipop two-dimensional (2D) gene plot depicting location of the pathogenic variants; size of the circle represents the proportion of individuals harboring the variant. Red circles indicate E198K and E420K missense variants. (C) RT-qPCR quantification of relative *PPP2R5D* mRNA levels in the hPSC lines. *PPP2R5D* transcript in the KO line is averaged at 7.3% ± 2.7% compared to the H9 control (*p* = 0.0001). Data are represented as the mean ± SD, and each point represents data from one independent biological replicate. *p* values: ns, not significant and ∗∗∗*p* < 0.0005.

Heterozygous missense variants in *PPP2R5D* cause the rare PPP2R5D-related neurodevelopmental disorder (NDD) Houge-Janssens Syndrome 1 (OMIM: 616355). PPP2R5D-related NDD is characterized by moderate to severe developmental delay and intellectual disability, autism spectrum disorder, attention deficits, seizures, and speech and motor impairments. A common feature of individuals with PPP2R5D-related NDD is macrocephaly.^2^^,^^3^^,^^4^^,^^5^ Some individuals with pathogenic *PPP2R5D* missense variants have exhibited early-onset Parkinson disease phenotypes that were responsive to levodopa treatment, suggesting that neurodegeneration may be part of the broader PPP2R5D disorder spectrum.^6^^,^^7^^,^^8^ To date, 16 *PPP2R5D* pathogenic or likely pathogenic variants have been observed in 111 published individuals (Figure 1A). Many pathogenic variants are recurrent *de novo* missense variants clustered in the highly conserved acidic loop that electrostatically interacts with substrates and is hypothesized to control PP2A phosphatase substrate specificity (Figure 1B).^9^^,^^10^ These missense variants change a negatively charged glutamic acid to a positively charged lysine, and reversal of these charged interactions may disrupt holoenzyme function and phosphatase substrate specificity. The c.592G>A (p.Glu198Lys) (GenBank: NM_006245.4) variant is the most common recurrent variant, accounting for nearly 50% of all individuals with PPP2R5D-related NDD, and is located in the acidic loop. The c.1258G>A (p.Glu420Lys) (GenBank: NM_006245.4) variant is located in another key region of the protein, which interacts with the catalytic subunit of the PP2A phosphatase and is observed in 7% of individuals. Notably, individuals with the E198K and E420K variants exhibit more severe neurodevelopmental phenotypes than those with other variants in *PPP2R5D*.^11^

A significant barrier to developing treatments for neurogenetic disorders is a limited understanding of the molecular and cellular pathogenic mechanisms. Patient-derived induced pluripotent stem cells (iPSCs) can be differentiated into diverse cell types, including neurons, and offer unprecedented insight into mechanisms associated with disease-causing mutations. This patient cell-specific model system enables investigation into the effects of specific pathogenic mutations in human neuronal cell types with the individual’s genomic background.

RNA-targeted therapeutics hold great promise for monogenic disorders for which the mutant allele is predicted to be dominant negative, and loss-of-function (LoF) alleles have no associated disease phenotype. Antisense oligonucleotides (ASOs) are single-stranded DNA gapmers that reduce target RNA levels by selectively binding to target RNA and inducing RNA cleavage via RNase H recruitment.^12^ Recently, ASOs have been designed that selectively silence dominant gene mutations associated with several neurological disorders and leave the functional allele of the target gene intact.^13^^,^^14^^,^^15^

To elucidate the pathogenesis of PPP2R5D-related NDDs, we have generated patient-derived iPSCs from individuals with the pathogenic E198K and E420K *PPP2R5D* variants, along with CRISPR-corrected isogenic controls, and differentiated them into neural progenitors and cortical glutamatergic neurons. Our studies reveal that pathogenic *PPP2R5D* variants cause increased neural progenitor proliferation and accelerated neurite outgrowth and lead to transcriptome-wide changes in key neuronal developmental and signaling pathways. Whether PPP2R5D-related NDD results from *PPP2R5D* gain of function or LoF is unclear, and we found that neuronal overgrowth phenotypes are not observed in neural progenitors and neurons lacking *PPP2R5D*. Furthermore, we have developed ASOs that selectively and efficiently reduce the E198K pathogenic transcript and rescue neurite outgrowth defects observed in patient iPSC-derived neurons.

## Material and methods

### CRISPR-Cas9 editing for hPSCs

This research was performed in compliance with relevant ethical regulations and approved by the Columbia University Medical Center Institutional Review Board (IRB #AAAT8830). E198K and E420K patient-derived iPSC lines, along with the corE198K isogenic control line, were obtained from the Simons Foundation Autism Research Initiative (SFARI IDs 15968-x1 and 15648-x2; clone IDs SV0003392, SV0003418, and SV000390). The patient-derived iPSC lines were reprogrammed from fibroblasts. Two isogenic pairs were generated in total using CRISPR-Cas9 gene editing by correcting the heterozygous variants c.592G>A (GenBank: NM_006245.4) (p.Glu198Lys) and c.1258G>A (GenBank: NM_006245.4) (p.Glu420Lys) in the patient-derived E198K and E420K iPSC lines, respectively.

The generation of the corE420K iPSC line was carried out in house using directed delivery of Cas9 and single-guide RNA (sgRNA) as a ribonucleoprotein complex using the previously described protocol.^16^ Briefly, a single sgRNA (GTCTGCCTCTCCCCACCAGG) and a donor DNA template (GAGTGAAATGAGCAGCACCCACCGAGTCTGCCTCTCCCCACCAGGTAGCAGAGCGTGCTCTCTATTACTGGAACAATGAGTACATCATG) were designed to specifically modify the *PPP2R5D* c.1258G>A mutation and electroporated into E420K iPSCs using the Lonza Nucleofector 4D system. Homology-directed repair efficiency was assessed by Inference of CRISPR Edits (ICE) analysis, and colonies of interest were selected and confirmed by Sanger sequencing.

The H9 embryonic pluripotent stem cell (ePSC) line was purchased from WiCell (WA09) and used to generate a homozygous *PPP2R5D* knockout (KO) ePSC line using CRISPR-Cas9 gene editing. Briefly, the KO line was generated by the delivery of Cas9 and two sgRNAs (CCCCACAGATTCGCCAACCC and AGATTCGCCAACCCAGGAGC) as a ribonucleotide complex. Clonal analysis identified a clone in which non-homologous end-joining repair resulted in a 5 bp deletion in exon 4 and the generation of an early termination codon.

### Maintenance of hPSC lines

Karyotype analysis of all human pluripotent stem cell (hPSC) lines was performed and determined to be normal. hPSCs were maintained in mTeSR Plus medium (STEMCELL Technologies) on plates pre-coated with Geltrex (Gibco) dissolved in DPBS without calcium or magnesium (Gibco) at a concentration of approximately 16 μg/mL. hPSCs were passaged as needed using Versene (Gibco). All cell lines were regularly tested for mycoplasma contamination and genotyped with Sanger sequencing.

### Immunofluorescence and microscopy

Cells were grown in 35 mm glass-bottom dishes affixed with no. 1.5 coverslips (Mattek P35G-1.5-14-C) coated with either Geltrex (hPSCs and neural stem cells [NSCs]) or poly-D-lysine and laminin (neurons) for microscopy purposes. Cells were fixed onto the coverslips at room temperature (RT) for 15 min in 4% PFA, followed by 3× 10 min washes in PBS. Coverslips were blocked in 1% bovine serum albumin and 5% goat serum in PBS with 0.1% Triton X-100 at RT for 45 min and incubated in primary antibody solution overnight at 4°C, followed by 3× 10 min washes in PBS. Cells were then incubated in secondary antibody solution at RT for 45 min, followed by 3× 10 min PBS washes. Cells were mounted in mounting media and imaged on a Zeiss LSM 710 or LSM 900 confocal microscope. For a list of antibodies used, see Table S1.

### Quantitative RT-PCR analysis

RNA was isolated from neurons from independent differentiations for each line in TRIzol Reagent (Thermo Scientific), and total mRNA was extracted using the standard TRIzol RNA extraction protocol and purified using the Zymo RNA-concentrator kit (Zymo). Up to 1 μg of RNA was reverse transcribed into cDNA using the Transcriptor First Strand cDNA Synthesis Kit (Roche Diagnostics). For quantitative RT-PCR (RT-qPCR), 10 ng of cDNA was amplified using gene-specific primers and LightCycler 480 SYBR Green I Master (Roche Diagnostics) on a LightCycler 480 real-time PCR machine (Roche Diagnostics). For each gene, at least two to three technical replicates and three biological replicates were assayed. To determine allele-specific expression in E198K patient-derived neurons, we used allele-specific locked nucleic probes. Each reaction was multiplexed with probes for E198, K198, and the housekeeping gene ACTB. Statistical significance was determined using an unpaired t test. For a list of gene-specific primers and allele-specific probes, see Table S2.

### Western analysis

hPSCs were grown to confluent layers and collected following Versene dissociation. Total protein was extracted using the NE-PER Nuclear and Cytoplasmic Extraction Reagents kit (Thermo Scientific) along with the Halt Protease and Phosphatase Inhibitor (Thermo Scientific) following the kit’s protocol. Protein concentration was determined using the Pierce BCA Protein Assay Kit (Thermo Scientific). 10 μg of each protein sample was separated by electrophoresis, and proteins were transferred onto an iBlot 2 Transfer Stack nitrocellulose membrane (Thermo Scientific). Membranes were blocked at RT for 1 h in Intercept TBS blocking buffer (LiCor) and then incubated overnight with PPP2R5D (1:1,000) and B-ACTIN antibodies (1:10,000). The next day, membranes were washed 3× for 10 min in TBS-Tween buffer and then incubated with secondary antibodies (LiCor IRDye 800CW goat anti-rabbit immunoglobulin [Ig]G and IRDye 680RD goat anti-mouse IgG; 1:20,000 dilution) for 1 h at RT, followed by 3× 10 min washes in TBS-Tween. Protein bands were visualized using the LiCor Odyssey Imaging System. Three biological replicates were assayed for each hPSC line.

### NSC induction and neuronal differentiation

hPSCs were induced into NSCs and expanded into neural progenitors using previously described protocols.^17^ Briefly, hPSCs were plated on Geltrex-coated plates in neural induction medium (NIM; Gibco) and treated with 5 μm ROCK inhibitor overnight. After 6–8 days, or when cells reached maximum confluency, the NSCs were dissociated into single cells with Accutase (STEMCELL Technologies) and expanded in neural expansion medium (Gibco). After four passages, NSCs were differentiated into neurons and plated on poly-D-lysine- and laminin-coated coverslips or plates in neurobasal medium supplemented with B27, 10 μm ascorbic acid, 1× GlutaMAX supplement, and CultureOne supplement (all Gibco). After 3–5 days, or once neural progenitors adopted a neuronal-like morphology with clear projections, B27 Plus Supplement was substituted for B27. The neuronal medium was changed 3 days a week by removing half of the medium and adding half fresh medium.

### NSC proliferation assays and cell cycle analysis

To assess the proliferation rate, isogenic pairs of NSCs were grown simultaneously and plated at 500,000 per 20 mm Geltrex-coated coverslip. When NSCs reached 30%–40% confluency, cells were fixed in 4% PFA at RT for 15 min, followed by 3× 10 min PBS washes. Fixed NSCs were co-immunostained with KI67 and SOX2 and immunocytochemistry (ICC) performed using the methods described above, and then the NSCs were imaged on a Zeiss LSM 710 confocal microscope. The percentages of KI67^+^ and SOX2^+^ cells were calculated using ImageJ (Fiji v.2.14.0) software.

To directly measure DNA synthesis, isogenic pairs of NSCs were grown simultaneously and plated at 250,000 per 20 mm Geltrex-coated coverslip. 3–4 days after plating, NSCs were incubated with 20 μm EdU for 90 min and then fixed and permeabilized according to the manufacturer’s protocol (Thermo Scientific). A Click-IT reaction was performed to detect EdU^+^ cells, and then they were washed and incubated with Hoescht dye for 30 min and analyzed on a Novocyte Quanteon Flow Cytometer. Cells in the G1 phase were identified as EdU^−^, Hoescht^low^; S phase cells were EdU^+^; and G2/M phase cells were EdU^−^, Hoescht^high^. Flow cytometry data were analyzed using the NovoExpress software.

### Neurite outgrowth assay

To assess neurite outgrowth, isogenic pairs of NSCs were differentiated into cortical neurons simultaneously and plated on 100,000 per 20 mm poly-D-lysine- and laminin-coated coverslips. At day *in vitro* (DIV)7, DIV14, DIV21, and DIV28, coverslips were fixed in 4% PFA at RT for 15 min, followed by 3× 10 min PBS washes. Fixed neurons were immunostained with MAP2 to identify neurites and ICC was performed using the methods described above, and the neurons were imaged on a Zeiss LSM 710 confocal microscope. Neurite lengths were measured using the Neuroanatomy package in ImageJ (Fiji v.2.14.0) software. Neurite lengths were calculated in two fields per coverslip for three independent differentiations for each line. A minimum of 130 neurites were measured for each line.

### iNGN2 differentiation of iPSCs

Patient-derived iPSCs were directly converted into induced cortical neurons (iNGN2) by forcing expression of the transcription factor NGN2 using a previously published protocol with minor changes.^18^ Human iPSCs were dissociated into single cells with Accutase (STEMCELL Technologies) and plated onto Geltrex-coated plates. On differentiation day −1, iPSCs were transduced with pLV-Ubi-rtTA and pLV-TetO-hNGN2-mCherry-Puro lentiviruses in the presence of polybrene (8 μg/mL, Sigma) overnight. Plasmids were provided by Ovid Therapeutics. The next day, NGN2 expression was induced using doxycycline (2 μg/mL, Millipore), and the medium was changed to NIM. On day 1, infected cells were then selected using puromycin (1 μg/mL, Invitrogen), and NIM was supplemented with doxycycline, and puromycin was changed every day for 3–4 days or until the culture was homogeneous with all cells containing neurites. Cells were then dissociated using Accutase and replated on poly-D-lysine- and laminin-coated coverslips in cortical neuron medium consisting of DMEM/F12, HEPES (Gibco), and neurobasal plus medium (Gibco) supplemented with B27 Plus Supplement (Gibco), BDNF (brain-derived neurotrophic factor; 10 ng/mL, Peprotech), GDNF (glial cell derived neurotrophic factor; 10 ng/mL, Peprotech), NT-3 (10 ng/mL, Peprotech), and 10 μm ascorbic acid (Sigma). Cortical neuron medium was changed 3 days a week by removing half of the medium and adding half fresh medium, and neurons were cultured for various durations until they were assayed as described.

### Bulk RNA sequencing and downstream analysis

Patient-derived iNGN2 neurons and isogenic controls were collected from three independent differentiations for each line at DIV14, and total mRNA was extracted. cDNA libraries were made for each individual neuronal sample using the TruSeq Stranded mRNA Library Prep Kit (Illumina), and 20 million pair-end reads were sequenced on a NovoSeq 6000 (Illumina). FASTQ files were aligned to the human reference genome (hg38) using STAR.^19^ Reads were counted using featureCounts, and differential expression analysis was performed for corE198K versus E198K neurons and corE420K and E420K neurons using DeSeq2.^20^^,^^21^ Volcano plots were generated using EnhancedVolcano software in RStudio.^22^ Gene Ontology (GO) term analysis was performed using Metascape software, and Qiagen Ingenuity Pathway Analysis (IPA) software was used for the canonical pathway analysis.^23^^,^^24^ RT-qPCR validation of target genes was performed on separate DIV14 neuronal samples collected from an additional three independent differentiations for all lines.

### ASO design and treatment

ASOs used in this study were produced by Integrated DNA Technologies. On day 3 of iNGN2 neuronal culture, ASO treatment was initiated by adding 10 μM ASOs in 500 μL of cortical neuron medium to the cells. 3 days after the initial ASO treatment, an additional 500 μL of medium was added, and the medium was changed three times a week by removing half of the medium and adding half fresh medium. E198K NSCs were treated with 10 μM ASOs 1 day after the cells were passaged and maintained in culture with the ASOs for 72 h. The required fresh medium was added to the culture at 48 h.

### Allele-specific expression analysis

To assess allele-specific expression following ASO treatment, corE198K, E198K untreated, and E198K ASO-treated iNGN2 neurons were collected from three independent differentiations for each line/treatment, and total mRNA was extracted and reverse transcribed into cDNA using the previously mentioned protocols. The *PPP2R5D* E198K region was amplified using primers with adapters and submitted to Azenta/GENEWIZ for Amplicon-EZ library preparation and sequencing. For each sample, reads containing either the E198 or K198 SNP were quantified, and the proportions of E198 and K198 reads were multiplied by total *PPP2R5D* expression to calculate the total remaining E198 and K198 allele expression.

### Statistical analysis

Data analyses were performed using Prism 10 software (GraphPad Software, USA). Statistical significance was evaluated by a two-tailed unpaired Student’s t test. For neurite outgrowth assays, we performed a one-way ANOVA test to determine between-group differences, and statistical differences between group means were determined using an independent sample t test. Statistical data were obtained from three independent neuronal differentiations for each line, and statistical significance was defined by *p* < 0.05 in all analyses.

## Results

### Generation of PPP2R5D hPSC lines

To investigate how pathogenic *PPP2R5D* variants impact neuronal development, we utilized two patient-derived iPSC lines, isogenic controls, and a *PPP2R5D* KO line. We obtained iPSCs derived from an individual with the highly recurrent E198K variant and an isogenic control in which the E198K allele has been corrected by CRISPR-Cas9 genome engineering (corE198K). We also obtained iPSCs from a separate individual carrying the E420K variant allele and used CRISPR-Cas9 genome editing to correct the E420K c.1258G>A variant and create an isogenic gene-corrected control line (corE420K) (Figure S1A).

Heterozygous missense variants are the main cause of PPP2R5D-related NDDs, and to date, there is no evidence that *PPP2R5D* LoF variants result in NDD or neurological disease. Population genomic data suggest that *PPP2R5D* has a high probability of being LoF intolerant (gnomAD probability of loss-of-function intolerance, pLI = 1).^25^^,^^26^^,^^27^ We identified thirteen individuals in the UK Biobank, thirteen individuals in the SPARK cohort, and eight individuals in the PCGC cohorts with twenty unique predicted LoF *PPP2R5D* variants predicted to lead to nonsense-mediated decay with frameshift or stop-gained variants.^26^^,^^27^^,^^28^ Individuals with predicted LoF *PPP2R5D* variants do not have any relevant neurological or developmental diagnoses reported, suggesting that *PPP2R5D* haploinsufficiency may be well tolerated.

To determine whether loss of *PPP2R5D* leads to pathological neuronal phenotypes compared to E198K and E420K variants, we also generated a *PPP2R5D* KO in the well-characterized and widely used H9 ePSC line using a CRISPR-mediated KO approach (Figure S1A).

We analyzed the three isogenic hPSC pairs (E198K and corE198K, E420K and corE420K, and H9 and *PPP2R5D* KO) for pluripotency markers and found no differences between any of the lines (Figures S1B and S1C). While the E198K and E420K variants had no effect on *PPP2R5D* gene or protein expression, *PPP2R5D* KO had almost complete loss of *PPP2R5D* expression (Figures 1C, S1D, and S1E).

### E198K and E420K NSCs exhibit increased proliferation

**Figure 2:**
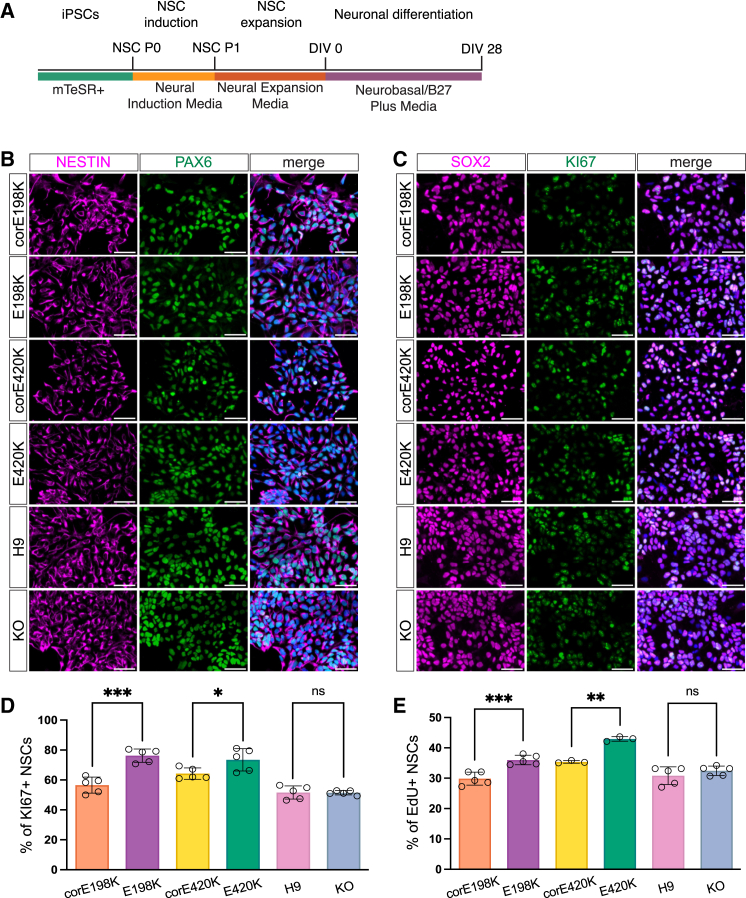
Induction of hPSCs revealed that E198K and E420K patient-derived NSCs are over-proliferative compared to controls (A) Schematic representation of the neural induction and neuronal differentiation protocol with timeline and media supplements. (B) Representative images of immunocytochemical characterization of the NSCs with NESTIN (magenta) and PAX6 (green). Scale bars: 50 μm. (C) Representative images of immunocytochemical detection of proliferating NSCs with SOX2 (magenta) and KI67 (green). Scale bars: 50 μm. (D) Quantification showing increased proliferative KI67^+^ in total SOX2^+^ neural progenitors in patient E198K and E420K NSCs. The number of biologically independent experiments is *n* = 5 per genotype. (E) Quantification showing increased EdU+ S-phase patient E198K and E420K NSCs. Data are represented as the mean ± SD, and each point represents data from one independent biological replicate. *p* values: ns, not significant, ∗*p* < 0.05, ∗∗*p* < 0.005, and ∗∗∗*p* < 0.0005.

The E198K and E420K patient-derived iPSCs and isogenic controls, along with the H9 and *PPP2R5D* KO cells, were induced into NSCs and analyzed for neural markers by immunostaining (Figure 2A). All lines were successfully induced into NSCs and exhibited high immunostaining of the neural progenitor markers SOX2, PAX6, and NESTIN (Figures 2B and 2C).

To investigate the effect of the E198K, E420K, and KO variants on neural progenitor proliferation, NSCs were immunostained with the cell proliferation marker KI67 (Figure 2C). NSCs differentiated from E198K and E420K patients had a significantly increased number of KI67^+^ cells compared to their respective isogenic controls, while there was no difference in the number of KI67 cells between the *PPP2R5D* KO and control lines (Figure 2D).

*PPP2R5D* has previously been shown to regulate genes that control cell cycle progression.^29^^,^^30^ Recently, the E198K and E420K variants were found to significantly increase mitotic duration and error rates in HEK cells.^31^ To further evaluate proliferation in patient-derived NSCs, we analyzed DNA synthesis by staining proliferating NSCs with EdU and Hoescht dye and determined the stages of the cell cycle via flow cytometry. E198K and E420K patient-derived NSCs have an increased proportion of NSCs in the S phase and G2/M phase and a decreased proportion of NSCs in the G1 phase (Figures 2E, S2A, and S2B). However, *PPP2R5D* KO NSCs do not exhibit cell-cycle-stage differences compared to the H9 control. These data suggest that the E198K and E420K variants disrupt cell cycle regulation, leading to an increased NSC proliferation rate.

### E198K and E420K PPP2R5D variants disrupt neurite outgrowth

**Figure 3:**
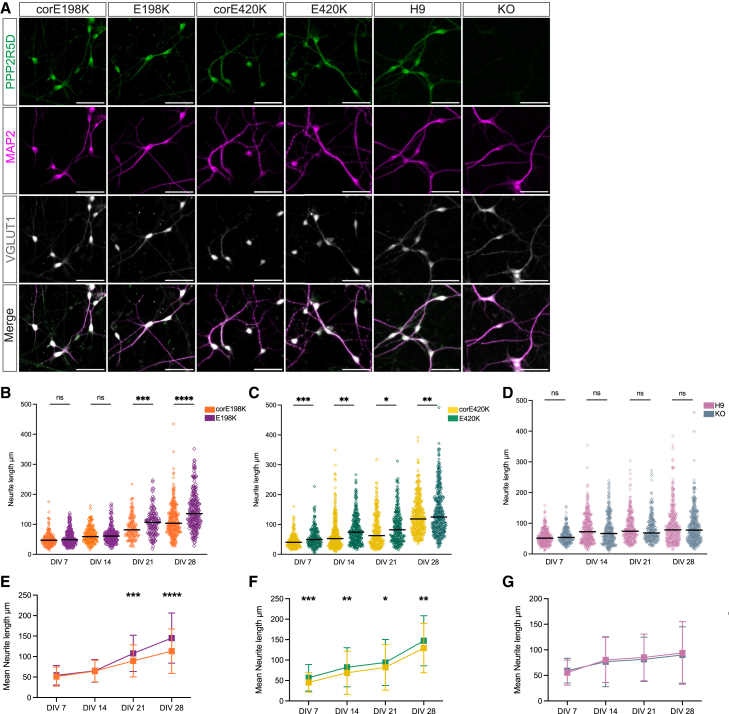
E198K and E420K patient-derived neurons exhibit accelerated neurite outgrowth (A) Representative images of immunocytochemical characterization of hPSC-derived neurons at DIV21 with PPP2R5D (green), MAP2 (magenta), and VGLUT1 (white). PPP2R5D immunostaining is near absent in the KO neurons. Scale bars: 50 μm. (B–D) These plots represent the neurite outgrowth analysis from each line. Each point represents the length of one individual MAP2^+^ neurite. The line represents the median neurite length. (E–G) These plots represent the mean neurite length with standard deviations, which are listed in Figure S3B. *p* values: ns, not significant, ∗*p* < 0.05, ∗∗*p* < 0.005, ∗∗∗*p* < 0.0005, and ∗∗∗∗*p* < 0.00005.

To determine the effect of pathogenic and LoF *PPP2R5D* variants on neuronal development, we differentiated the three isogenic pairs of NSCs into cortical glutamatergic neurons. Seven days post-differentiation (DIV7), neurons from all lines strongly expressed MAP2, indicating successful neuronal differentiation (Figure S3A). Our differentiation protocol predominately generates cortical glutamatergic neurons, and by DIV21, all MAP2^+^ cells co-expressed VGLUT1 (Figure 3A). PPP2R5D protein is predominately localized in the neuronal soma and neurites and is absent in the *PPP2R5D* KO neurons (Figure 3A). The E198K and E420K variants did not appear to alter PPP2R5D protein localization as compared to isogenic controls.

To investigate whether neuronal development is altered in patient-derived neurons or *PPP2R5D* KO neurons, we performed a time-course analysis of neurite outgrowth from DIV7 to DIV28 and measured the length of MAP2^+^ neurites every 7 days. E198K patient-derived neurons have significantly longer neurites by DIV21 compared to the corE198K isogenic control; by analyzing both the mean and median neurite length at each time point, this persists to DIV28 (mean neurite length DIV7: corE198K 51.1 + 23.3 μm, E198K 54.2 + 23.7 μm, *p* value is not significant; DIV14: corE198K 64.5 + 26.5 μm, E198K 65.1 + 27.8 μm, *p* value is not significant; DIV21: corE198K 89.4 + 39.1 μm, E198K 107.8 + 44.2 μm, *p* = 0.0004; and DIV28: corE198K 113.3 + 54.2 μm, E198K 145.1 + 61.4 μm, *p* < 0.0001) (Figures 3B and 3E). E420K patient-derived neurons are significantly longer compared to the isogenic control as early as DIV7 through DIV28, suggesting that E420K patient-derived neurons exhibit accelerated neurite outgrowth compared to the isogenic control (mean neurite length DIV7: corE420K 45.4 + 23.7 μm, E420K 56.8 + 32.5 μm, *p* = 0.0001; DIV14: corE420K 68.9 + 52.8 μm, E420K 82.5 + 47.8 μm, *p* = 0.0011; DIV21: corE420K 82.2 + 55.8 μm, E420K 94.2 + 56.4 μm, *p* = 0.0242; and DIV28: corE420K 129.2 + 60.6 μm, E420K 147.3 + 61.4 μm, *p* = 0.0031) (Figures 3C and 3F). The *PPP2R5D* KO line does not exhibit neurite outgrowth defects when compared to H9 control neurons (mean neurite length DIV7: H9 55.3 + 24.5 μm, KO 59.3 + 21.2 μm; DIV14: H9 80.3 + 44.2 μm, KO 76.7 + 48.8 μm; DIV21: H9 85.3 + 45.5 μm, KO 81.5 + 43.3 μm; and DIV28: H9 93.4 + 61.3 μm, KO 89.9 + 55.1 μm; all *p* values are not significant) (Figures 3D and 3G). These results suggest that the E198K and E420K variants cause a neurite outgrowth defect through a mechanism that is not LoF.

### Transcriptomic changes induced by pathogenic variants in patient-derived neurons

**Figure 4:**
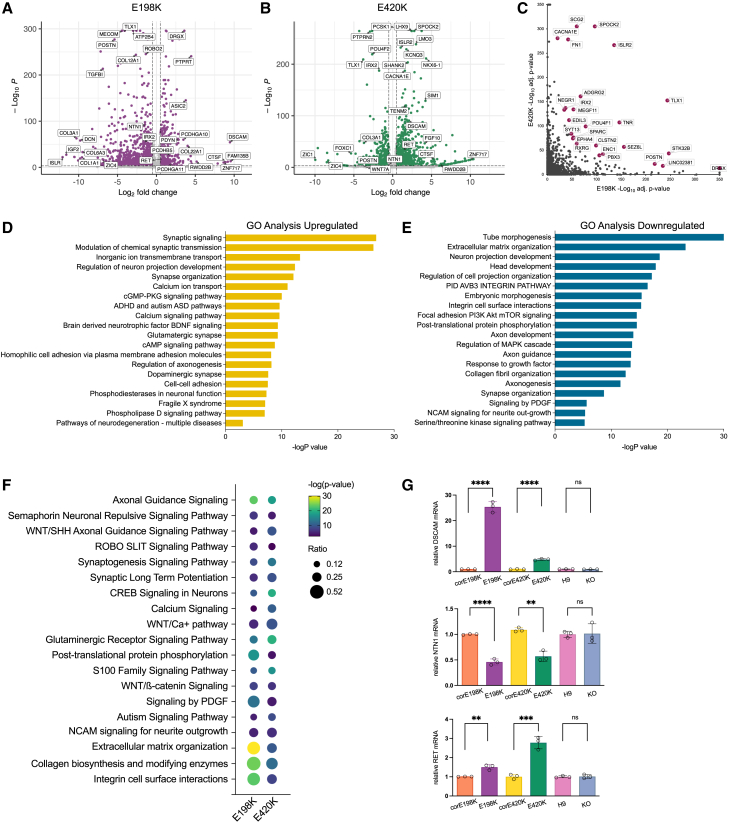
Transcriptional changes in patient-derived neurons reveal alterations in neuronal development and signaling pathways (A and B) Volcano plot of FDR adjusted *p* values versus log_2_ fold change for differential expression analysis between the isogenic and patient-derived iNGN2 glutamatergic neurons. DEGs are highlighted in purple for E198K and green for E420K and are based on false discovery rate (FDR) < 1% and log fold change > ±0.5. (C) Plot of the FDR-adjusted *p* values for the E420K analysis versus the E198K analysis with the top 25 significantly DEGs highlighted in red. (D and E) Bar graph of Gene Ontology analysis of the shared DEGs in E198K and E420K neurons. The enriched Gene Ontology terms are denoted on the left, and the *x* axis represents the −log_10_ of the *p* value. (F) The 1,763 DEGs from the E198K neurons and the 1,986 DEGs from the E420K neurons were used as input for the Ingenuity Pathway Analysis (IPA). The size of the dot represents the proportion of genes from each DEG dataset divided by the total number of genes making up that pathway in the IPA reference database mapped to the canonical pathway. The color of the dot represents the log_10_*p* value. (G) RT-qPCR quantification of relative mRNA levels from three independent neuronal differentiations. Data are represented as the mean ± SD. *p* values: ∗∗*p* < 0.005 and ∗∗∗∗*p* < 0.00005.

RNA sequencing (RNA-seq) was performed on iPSC-derived glutamatergic neurons at DIV14 to further characterize the molecular effects of the E198K and E420K variants. To ensure a homogeneous population of glutamatergic neurons, we directly induced neuronal differentiation by transfecting iPSCs with lentivirus vectors overexpressing *NGN2* using previously established protocols (Figure S4A).^18^ Direct reprogramming of iNGN2 neurons allowed for rapid induction of a pure population of glutamatergic neurons (Figure S4B). A total of 1,763 differentially expressed genes (DEGs) were observed between the E198K patient-derived and the isogenic corE198K neurons (1,287 downregulated and 476 upregulated), and a total of 1,986 DEGs were observed between the E420K patient-derived and isogenic corE420K neurons (600 downregulated and 1,386 upregulated; DEGs were defined as an adjusted *p* value <0.05 and log_2_ fold-change > ±0.5; Figures 4A and 4B).

The E198K and E420K lines shared 351 DEGs, with 147 upregulated and 204 downregulated in the patient-derived neurons compared to their respective isogenic controls (Figure 4C). GO term analysis of the shared DEGs revealed pathway enrichment of neuronal development and function, including synaptic signaling and synapse development, axonogenesis and axonal guidance, and neurite outgrowth, and several signaling pathways important for neuronal development, including BDNF, cAMP, PI3K-AKT-mTOR, and PDGF (Platelet Derived Growth Factor) (Figures 4D and 4E). Several regulators of cell adhesion were misregulated, suggesting a disruption of neuronal adhesion in the patient-derived neurons. IPA canonical pathway analysis revealed aberrant regulation of key signaling pathways involved in axonal guidance, synaptogenesis, calcium signaling, and intracellular signal transduction, suggesting a cascade of developmental and functional impairments in the patient-derived neurons as a result of pathogenic E198K and E420K variants (Figure 4F). Dysregulation of pathways important for neurite outgrowth and structural integrity, such as NCAM signaling, extracellular matrix organization, collagen biosynthesis, and integrin cell surface interactions, was found. Additionally, there were significant disruptions in WNT signaling, suggesting that *PPP2R5D* pathogenic variants disrupt developmental cues in patient-derived neurons.

This transcriptomic analysis suggests that important regulators of neurite outgrowth may be altered in the E198K and E420K patient-derived neurons. We confirmed differential expression of a subset of genes in independent batches of neurons by RT-qPCR analysis. *DSCAM* is a cell adhesion molecule important for neuronal development and neurite outgrowth, and notably, *DSCAM* overexpression is implicated in many NDDs.^32^^,^^33^^,^^34^^,^^35^^,^^36^ *DSCAM* was the most significantly upregulated gene in E198K neurons, with E198K patient neurons exhibiting approximately 25-fold increased expression compared to corE198K isogenic neurons and 5-fold in E420K neurons (Figure 4G). *DSCAM* is a receptor for *NTN1*, a secreted ligand important for neurite outgrowth and axon guidance, and *NTN1* expression is significantly decreased in both E198K and E420K patient neurons compared to isogenic controls (Figure 4G).^37^^,^^38^ *RET* is a receptor tyrosine kinase that promotes cell growth, and *RET* activation has been shown to accelerate neurite outgrowth.^39^ *RET* is significantly upregulated in E198K and E420K patient neurons (Figure 4G). *DSCAM*, *NTN1*, and *RET* expression is not changed in *PPP2R5D* KO neurons, suggesting that *PPP2R5D* LoF does not affect neurite outgrowth or neuronal development as significantly as the E198K and E420K variants. Altogether, these findings indicate that the E198K and E420K variants alter key pathways involved in neurite outgrowth, axon guidance, and neuronal development in patient-derived glutamatergic neurons.

### Allele-specific ASO design to selectively knock down the E198K allele

**Figure 5:**
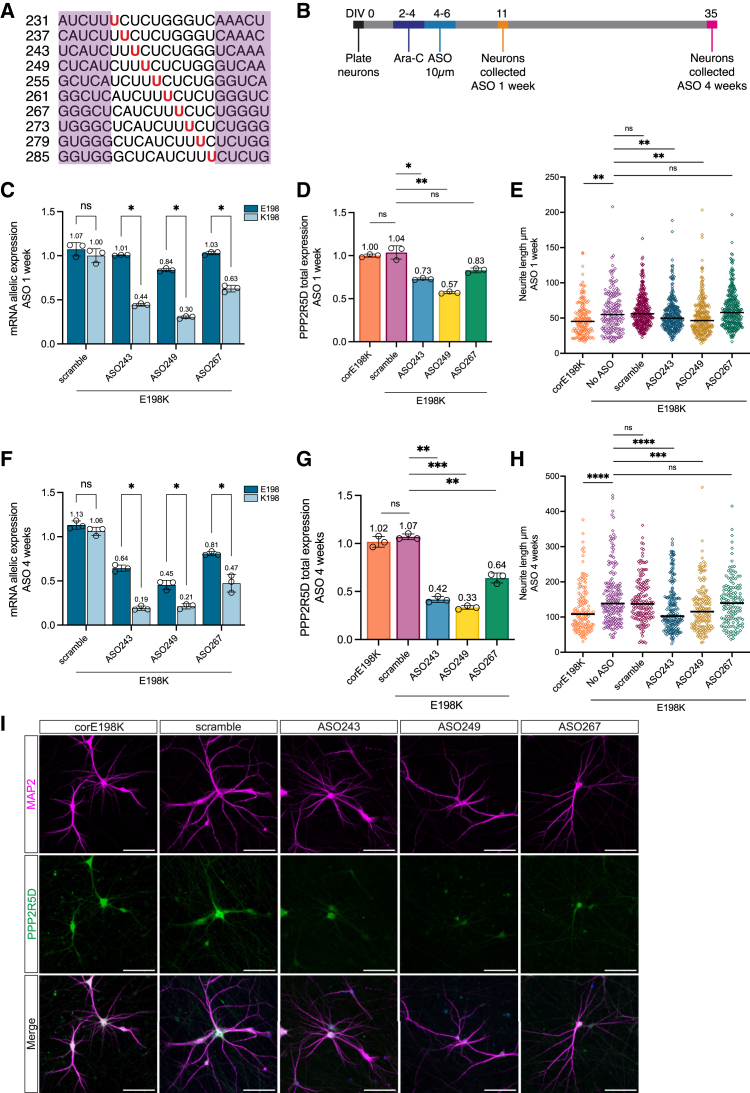
Selective ASO knockdown of the K198 mutant allele rescues neurite outgrowth defects in E198K patient-derived neurons (A) ASO gapmers with targeted c.592G>A (p.E198K) SNPs shown in red. 2′MOE modifications on the external 5 base pairs are highlighted in purple boxes. (B) Timeline of ASO treatment of iNGN2 neurons. (C) Allele-specific mRNA expression of E198K patient-derived DIV11 iNGN2 neurons treated with ASOs for 1 week. (D) RT-qPCR quantification of relative *PPP2R5D* mRNA levels in E198K DIV11 iNGN2 neurons treated with ASOs for 1 week. (E) These plots represent the neurite outgrowth analysis at DIV11 of E198K iNGN2 neurons treated with ASOs for 1 week, along with the corE198K isogenic control. Each dot represents the length of an individual MAP2^+^ neurite. The line represents the median neurite length: corE198K 45.3 μm, E198K no ASO 55 μm, scramble ASO 56.1 μm, ASO243 29.8 μm, ASO249 46.8 μm, and ASO267 57.9 μm. (F) Allele-specific mRNA expression of E198K patient-derived DIV35 iNGN2 neurons treated with ASOs for 4 weeks. (G) RT-qPCR quantification of relative *PPP2R5D* mRNA levels in E198K DIV35 iNGN2 neurons treated with ASOs for 4 weeks. (H) These plots represent the neurite outgrowth analysis at DIV35 of E198K iNGN2 neurons treated with ASOs for 4 weeks, along with the corE198K isogenic control. Each point represents the length of an individual MAP2^+^ neurite. The line represents the median neurite length: corE198K 108.3 μm, E198K no ASO 138.5 μm, scramble ASO 140.7 μm, ASO243 102.7 μm, ASO249 115.3 μm, and ASO267 139.7 μm. (I) Representative images of immunocytochemical detection of MAP2 (magenta) and PPP2R5D (green) in E198K DIV35 iNGN2 neurons treated with ASOs, along with the corE198K isogenic control. Scale bars: 100 μm. Data are represented as the mean ± SD. *p* values: ns, not significant, ∗*p* < 0.05, ∗∗*p* < 0.005, ∗∗∗*p* < 0.0005, and ∗∗∗∗*p* < 0.00005.

Given that LoF *PPP2R5D* variants are not associated with disease and pathogenic *PPP2R5D* mutations are predicted to negatively alter protein function, we hypothesize that allele-specific reduction of the pathogenic *PPP2R5D* transcript in patient-derived neurons will rescue neurite outgrowth defects. We designed ASOs that selectively target the K198 mutant allele while preserving expression of the reference E198 wild-type allele. We designed 20-nucleotide gapmer ASOs with fully phosphorothioate (PS)-modified backbones and 2′ methoxy-ethyl (MOE)-modified bases at both ends. To develop ASOs with the greatest discrimination between K198 and E198 and the strongest knockdown of the K198, we placed the targeted nucleotide at various positions in the internal bases (Figure 5A). We performed a pilot experiment in E198K iNGN2 neurons by treating E198K neurons with ASOs by gymnotic uptake at a 10 μm final concentration for 72 h. We observed the greatest specificity in RNA reduction between the E198 and K198 alleles when the targeted nucleotide was placed in the central region of the ASO, and candidates ASO243, ASO249, and ASO267 were selected for further analysis (Figure S4C). Treatment of E198K NSCs with ASOs for 72 h did not result in significant knockdown, likely due to the highly proliferative nature of the NSCs leading to rapid dilution of the ASO among daughter cells (Figures 2D, 2E, and S4D).

### Selective reduction of the E198K pathogenic allele rescues neurite overgrowth

E198K patient-derived iNGN2 neurons were treated with a single ASO dose from DIV4 through DIV6. Neurons were processed for RNA extraction and fixed for neurite outgrowth analysis on DIV11 and DIV35, 1 and 4 weeks post-ASO treatment, respectively (Figure 5B). We performed next-generation amplicon sequencing on RNA extracted from ASO-treated iNGN2 neurons to quantify the remaining E198 and K198 transcripts.

After 1 week of treatment with ASO243, ASO249, and ASO267, the E198K neurons exhibited significant knockdown of the K198 mutant allele (56%, 70%, and 37%, respectively) and minimal knockdown of the E198 allele (0%, 16%, and 4%, respectively) compared to a non-targeting scramble ASO control (Figure 5C). We also quantified *PPP2R5D* expression via RT-qPCR and found that compared to the E198K neurons treated with the scramble ASO control, ASO243, ASO249, and ASO267 exhibit 27%, 43%, and 17% knockdown of total *PPP2R5D* (Figure 5D). Our results suggest that at DIV11, most of the reduction of *PPP2R5D* expression is due to K198 transcript knockdown.

By DIV11, E198K iNGN2 neurons begin to exhibit significantly longer neurite lengths compared to corE198K isogenic controls (corE198K mean: 49.33 ± 24.54 μm; E198K mean: 59.04 ± 29.80 μm; Figure 5E). E198K neurons treated with the scrambled ASO control show no difference in neurite outgrowth compared to untreated E198K neurons (scramble ASO control mean: 60.78 ± 22.76 μm; Figure 5E). E198K neurons treated with ASO243 and ASO249 have a shorter mean neurite length compared to neurons treated with the scramble control, although not significant for ASO243 at this time point (ASO243 mean: 54.78 ± 23.25 μm; ASO249 mean: 52.37 ± 26.73 μm). E198K neurons treated with ASO267 exhibit no change in neurite length compared to neurons treated with the scramble control (ASO267 mean: 62.68 ± 26.27 μm).

After 4 weeks of treatment with ASO243, ASO249, and ASO267, we observed increased knockdown of the K198 mutant allele (81%, 79%, and 53%, respectively), although we also observed more knockdown of the E198 allele (36%, 55%, and 19%, respectively) compared to the scramble ASO control (Figure 5F). Total *PPP2R5D* expression is also significantly reduced in E198K neurons treated with ASO243, ASO249, and ASO267 (58%, 67%, and 36%, respectively) (Figure 5G). We confirmed that PPP2R5D protein is also reduced in the ASO-treated neurons by immunostaining, while MAP2 expression remains unchanged (Figure 5I). While we observed a more significant reduction of the E198 allele at DIV35 compared to DIV11, ASO243, ASO249, and ASO267 exhibit more selective knockdown of the K198 allele compared to the E198 allele at both time points.

At DIV35, E198K patient-derived iNGN2 neurons have significantly longer neurites than corE198K isogenic control neurons, and treatment of E198K neurons with the scramble ASO control had no effect on neurite length (corE198K mean: 123.9 ± 67.24 μm; E198K mean: 148.5 ± 81.33 μm; scramble ASO control mean: 149.3 ± 67.60; Figure 5E). After 4 weeks of treatment with ASO243 and ASO249, we observed complete rescue of the neurite overgrowth phenotype in E198K neurons, with significant reduction of neurite lengths compared to neurons treated with the scrambled ASO control (ASO243 mean: 126.9 ± 68.65 μm; ASO249 mean: 127.1 ± 66.87 μm; Figure 5H). E198K neurons treated with ASO267 did not exhibit neurite length reduction (ASO267 mean: 143.1 ± 67.93; Figure 5H). This is perhaps not surprising, given that ASO267 treatment was less effective at reducing total *PPP2R5D* levels overall and was less allele specific. These findings suggest that selective knockdown of the K198 pathogenic allele is sufficient to rescue pathologic neurite outgrowth defects observed in E198K patient neurons *in vitro*.

## Discussion

Here, we investigate the impact of *PPP2R5D* pathogenic variants on developing neurons derived from patients and offer insight into the molecular disease mechanisms of the rare PPP2R5D-related NDD. Additionally, we provide evidence that knockdown of the E198K pathogenic variant is sufficient to rescue neurite outgrowth defects observed in patient-derived neurons.

Recent advances in genome sequencing have accelerated the identification of genomic variants underlying rare NDDs, yet treatment remains a significant challenge. A key barrier to therapeutic development is the limited understanding of NDD etiology and the mechanisms driving these disorders. Pathogenic *PPP2R5D* missense variants are thought to alter PP2A phosphatase target specificity, disrupting downstream pathways critical for neuronal development and maintenance. However, the precise mechanisms linking these variants to observed patient phenotypes remain unclear. Phosphoproteomic studies have shown that the E198K and E420K variants lead to increased phosphorylation of several PPP2R5D substrates, including ribosomal protein S6 and AKT-mTOR signaling cascades in HEK293 cells, suggesting these variants alter the PPP2R5D-dependent PP2A activity by affecting substrate specificity.^30^^,^^40^ Additionally, the E198K variant has been shown to affect dopamine pathways and neuronal morphology in midbrain neurons derived from patient iPSCs.^41^ These findings indicate that *PPP2R5D* variants disrupt neuronal development and signaling, contributing to NDD pathogenesis.

Our study identifies *PPP2R5D* as a potential regulator of neurite outgrowth and neural progenitor proliferation, representing a potential disease mechanism for the brain overgrowth commonly observed in individuals with *PPP2R5D* pathogenic variants. Notably, neuronal overgrowth is not observed in neurons lacking *PPP2R5D*, suggesting that the pathogenic missense variants change PPP2R5D protein function and substrate specificity as opposed to loss or reduced function of *PPP2R5D*. Disrupted neurite outgrowth in patient-derived glutamatergic neurons may result from altered pathways critical for neuronal development, function, and structural integrity. Our transcriptomic analysis revealed that pathogenic *PPP2R5D* variants may disrupt neuronal processes by affecting cell signaling cascades essential for axonal guidance, extracellular matrix interactions, and WNT signaling, leading to impaired neuronal development. Additionally, changes in synaptogenesis, glutamatergic signaling, and calcium signaling suggest deficits in synaptic transmission, plasticity, and neuronal communication. Future studies should evaluate the functional activity of patient-derived neurons, and proteomic analyses will be important for identifying the PP2A-PPP2R5D substrates involved in neurodevelopment and neuronal function.

Many of the cellular and molecular processes critical for early brain and neuronal development are also required to maintain proper neuronal function and brain health into adulthood. Early-onset NDDs are increasingly associated with neurodegeneration, and many proteins and pathways that play roles in nervous system development are also implicated in neurodegenerative disorders.^42^^,^^43^^,^^44^ Recently, the recurrent pathogenic *PPP2R5D* variants E198K and E200K have been associated with early-onset parkinsonism. Three individuals with the E200K *PPP2R5D* variant and one individual with the E198K variant presented with developmental delay, intellectual disability, seizures, and brain overgrowth features and developed progressive motor decline and levodopa-responsive parkinsonism between the ages of 25 and 40 years.^6^^,^^7^^,^^8^ Postmortem examination of one individual demonstrated partial loss of substantia nigra neurons in the absence of Lewy body pathology. These findings demonstrate a link between *PPP2R5D* pathogenic variants and neurodegeneration, highlighting the potential importance that therapeutic treatment for *PPP2R5D*-related NDD will have long-lasting effects and may curb early-onset parkinsonism.

For monogenic neurodevelopmental and neurodegenerative disorders caused by recurrent pathogenic missense variants, particularly for disorders in which LoF is not associated with disease, allele-specific ASOs represent an ideal therapeutic approach. Targeted reduction of the pathogenic allele presents a potential avenue for restoring normal function through expression of the remaining wild-type allele. We developed an ASO therapeutic strategy that reduced the transcript from the K198 pathogenic allele by 81% in E198K patient-derived neurons and preserved 64% of the E198 wild-type allele transcript (ASO243). E198K patient-derived neurons treated with the most selective ASOs (ASO243 and ASO249) rescued neurite overgrowth phenotypes, while neurons treated with the less efficacious ASO267 (which reduced expression of the K198 allele by 53%) did not rescue neurite overgrowth. Utilizing an ASO-mediated RNA knockdown approach results in the loss of *PPP2R5D*; however, our findings that neurons lacking *PPP2R5D* do not display neural progenitor hyperproliferation or neurite outgrowth deficits suggest that haploinsufficiency may be tolerated. These findings suggest that *PPP2R5D* knockdown is tolerable in neurons *in vitro*; however, a high level of pathogenic allele knockdown must be achieved in order to reverse disease-associated phenotypes.

## Data and code availability

The accession number for the RNA-seq FASTQ and processed files reported in this paper are available at the NIH NCBI GEO: GSE266958.

## Acknowledgments

We thank our family partners and research participants, the Jordan’s Guardians Angels Foundation, and members of the Chung lab, including Bryan Diaz and Amanda McPartland, for their contributions to this project. Funding for this project was provided to W.K.C. by Ovid Therapeutics and UCAL CU17-2559 and to R.E.Y. by the CTSA TL1 postdoctoral fellowship #TL1TR001875. hPSC gene editing experiments were performed in the Columbia Stem Cell Facility at Columbia University Irving Medical Center under the leadership of Barbara Corneo, PhD, and staff Grazia Iannello, PhD, and Achchhe Patel, PhD. Flow cytometry experiments were performed in the Columbia Stem Cell Initiative Flow Cytometry core facility at Columbia University Irving Medical Center under the leadership of Michael Kissner. RNA-seq was conducted at the Boston Children’s Hospital Molecular Genetics Core.

## Author contributions

All authors contributed to the interpretation of results and the final version of the manuscript. R.E.Y., N.D.G., T.H.K., P.S., and W.K.C. conceived and designed the study. R.E.Y., M.V.Z., and C.A.L. collected the data and performed the analyses. R.E.Y. and W.K.C. drafted and wrote the manuscript.

## Declaration of interests

W.K.C. has received research funding from Ovid Therapeutics. N.D.G., T.H.K., and P.S. are current and former employees of Ovid Therapeutics.

## Web resources

GenBank, https://www.ncbi.nlm.nih.gov/genbank/
OMIM, https://www.omim.org
NIH NCBI GEO, https://www.ncbi.nlm.nih.gov/geo/

## Supplemental information

Document S1. Figures S1–S4 and Tables S1 and S2

Document S2. Article plus supplemental information
