# Supplementary material for: Pathogenic PPP2R5D variants disrupt neuronal development and neurite outgrowth in patient-derived neurons that are reversed by allele-specific knockdown
Source: HGG Adv. 2025 May 8;6(3):100450. doi: 10.1016/j.xhgg.2025.100450 (PMC12148737; doi:10.1016/j.xhgg.2025.100450)
Supplement: Document S1. Figures S1–S4 and Tables S1 and S2 [file mmc1.pdf]

HGGA, Volume *6*

Supplemental information

Pathogenic PPP2R5D variants disrupt neuronal development and neurite outgrowth in patient-derived neurons that are reversed by allele-specific knockdown

Randee E. Young, Michael V. Zuccaro, Charles A. LeDuc, Noelle D. Germain, Tae Hyun Kim, Patrick Sarmiere, and Wendy K. Chung

Supplemental Information

Supplemental Figures and Legends

Figure S1

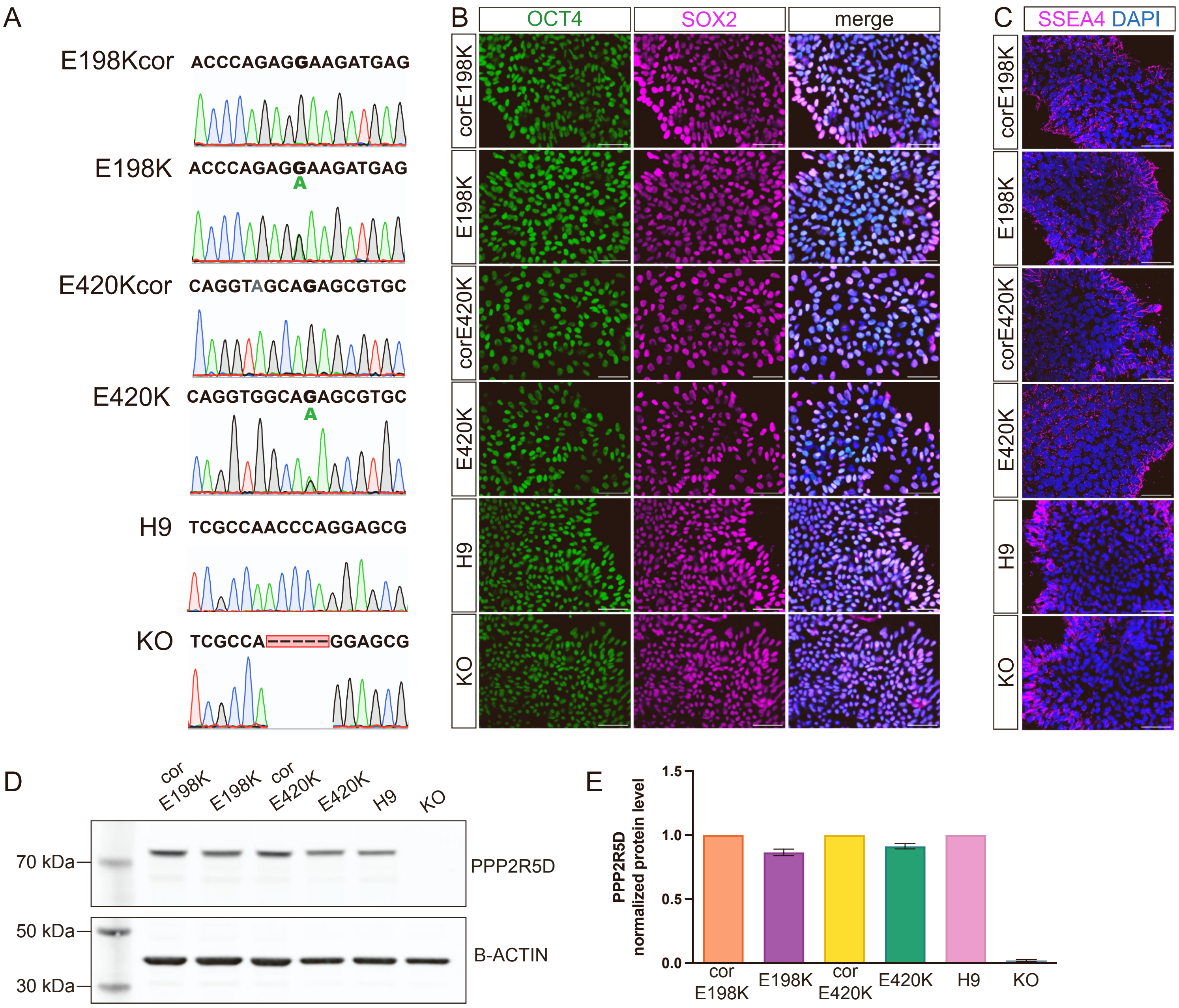
A
E198Kcor ACCCAGAGGAAGATGAG
E198K ACCCAGAGGAAGATGAG
A
E420Kcor CAGGTAGCAGAGCGTGC
E420K CAGGTGGCAGAGCGTGC
A
H9 TCGCCAACCCAGGAGCG
KO TCGCCA-----GGAGCG
B
OCT4
SOX2
merge
corE198K
E198K
corE420K
E420K
H9
KO
C
SSEA4 DAPI
corE198K
E198K
corE420K
E420K
H9
KO
D
cor E198K
E198K
cor E420K
E420K
H9
KO
70 kDa
50 kDa
30 kDa
PPP2R5D
B-ACTIN
E
PPP2R5D normalized protein level
1.5
1.0
0.5
0.0
cor E198K
E198K
cor E420K
E420K
H9
KO

Figure S1

A) Sanger sequencing plot of each iPSC line depicting the pathogenic and KO mutations with corrected controls. B) Representative images of immunocytochemical detection of OCT4 (green) and SOX2 (magenta); and C) SSEA4 (magenta) in hPSCs. Scale bars: 50 µm. D) Western analysis of total cell lysates immunostained for PPP2R5D and B-ACTIN. E) Quantification of relative amounts of PPP2R5D normalized to B-ACTIN and relative to controls for each line. Data are represented as the mean ± SD.

Figure S2

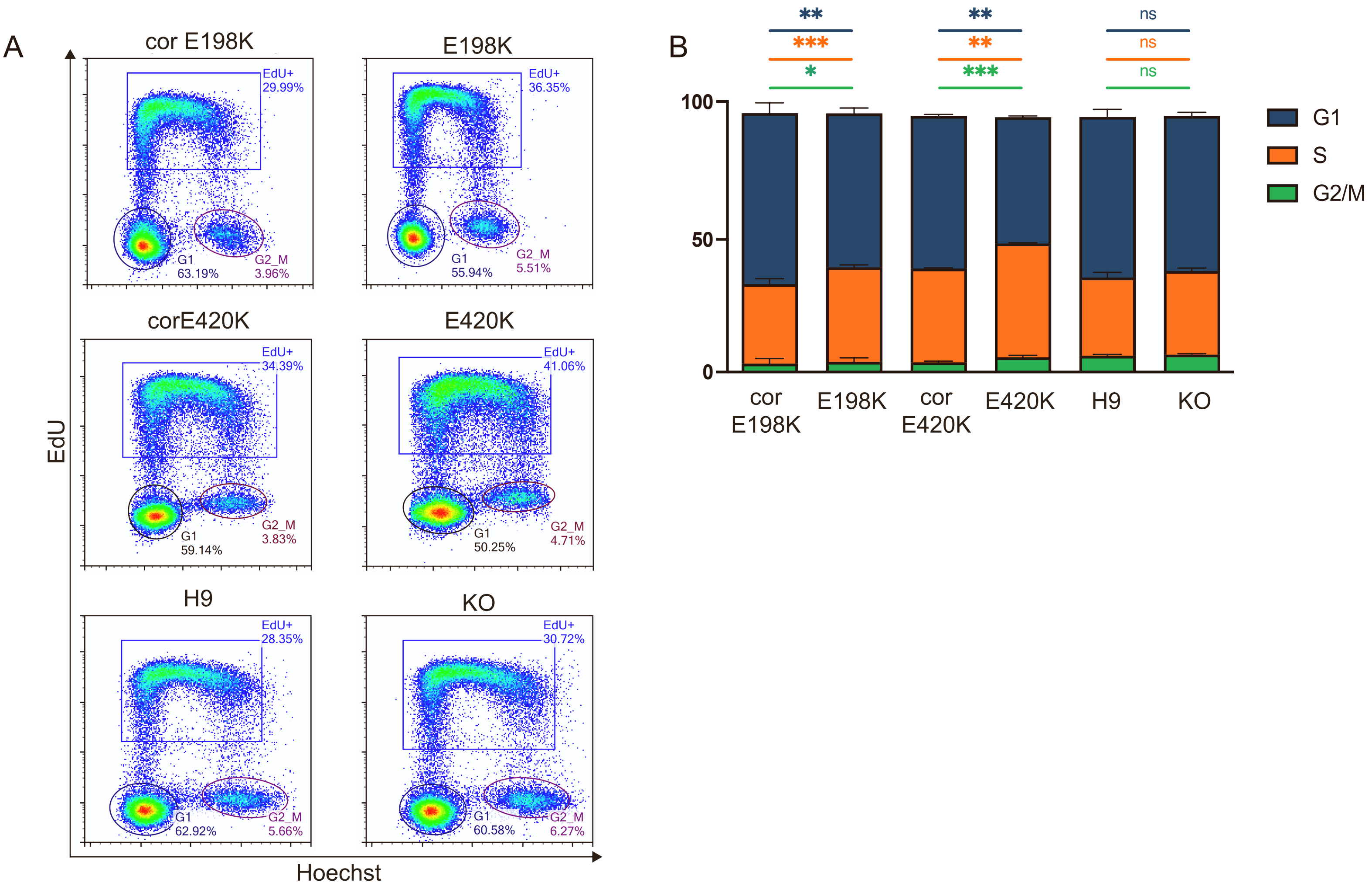
A
cor E198K
EdU+
29.99%
G1
63.19%
G2_M
3.96%
E198K
EdU+
36.35%
G1
55.94%
G2_M
5.51%
corE420K
EdU+
34.39%
G1
59.14%
G2_M
3.83%
E420K
EdU+
41.06%
G1
50.25%
G2_M
4.71%
H9
EdU+
28.35%
G1
62.92%
G2_M
5.66%
KO
EdU+
30.72%
G1
60.58%
G2_M
6.27%
EdU
Hoechst
B
**
***
*
**
**
***
ns
ns
ns
100
50
0
cor E198K
E198K
cor E420K
E420K
H9
KO
G1
S
G2/M

Figure S2

A) Flow cytometry plots of cell cycle analysis depicting Hoescht (X axis) and EdU (Y-axis). B) Bar graph representing the quantification of G1, S, and G2/M cell cycle phases for each NSC line. Data are represented as the mean $\pm$ SD and each point represents data from one independent biological replicate. p-values ns: not significant, *: $p<0.05$, **: $p<0.005$, ***: $p<0.0005$.

Figure S3

A

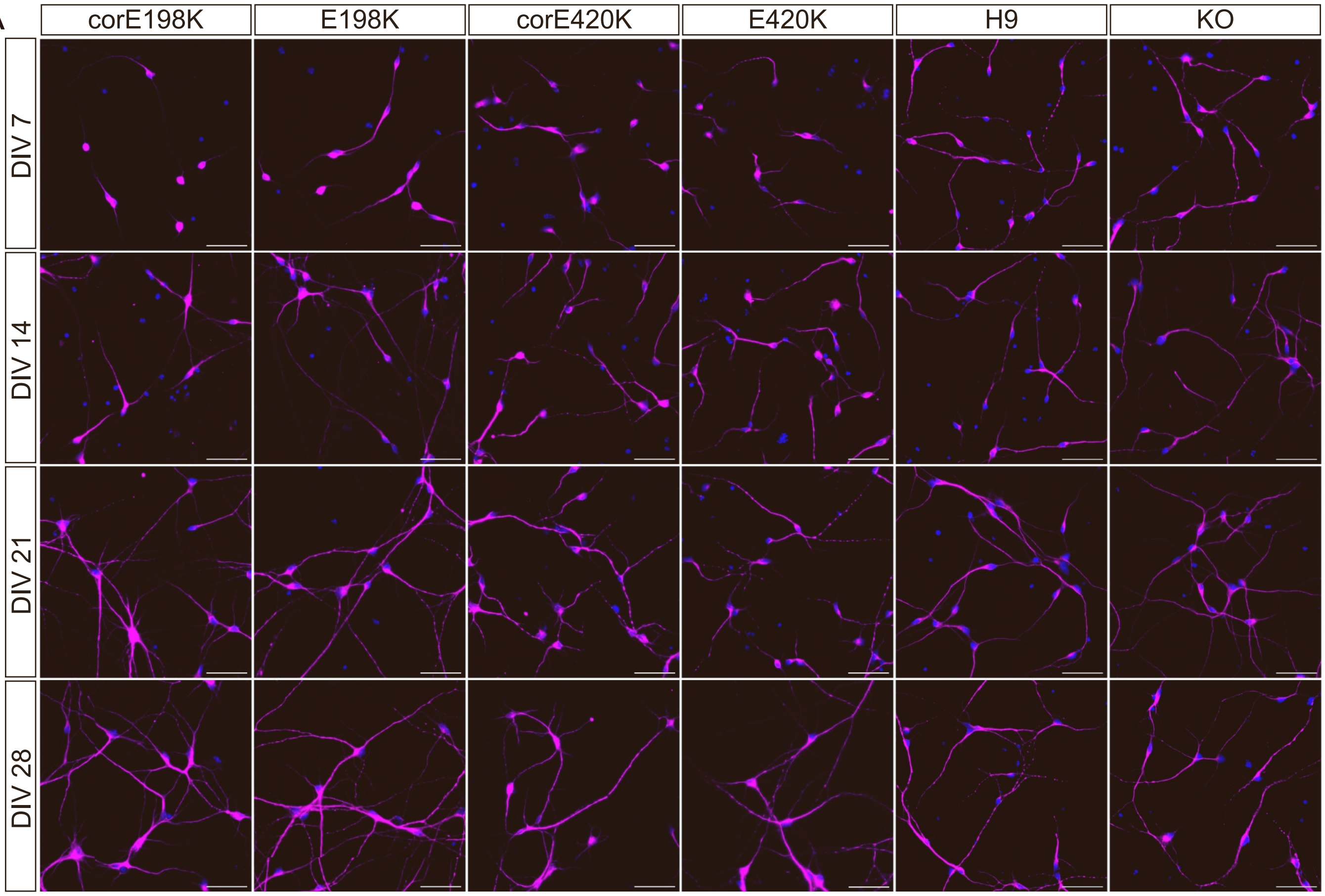

B

	corE198K	E198K	corE420K	E420K	H9	KO
DIV 7	51.1 + 23.3 μm	54.2 + 23.7 μm	45.4 ± 23.7 μm	56.8 ± 32.5 μm	55.3 ± 24.5 μm	59.3 ± 21.2 μm
DIV 14	64.5 + 26.5 μm	65.1 ± 27.8 μm	68.9 ± 52.8 μm	82.5 ± 47.8 μm	80.3 ± 44.2 μm	76.7 ± 48.8 μm
DIV 21	89.4 + 39.1 μm	107.8 + 44.2 μm	82.2 ± 55.8 μm	94.2 ± 56.4 μm	85.3 ± 45.5 μm	81.5 ± 43.3 μm
DIV 28	113.3 + 54.2 μm	145.1 + 61.4 μm	129.2 ± 60.6 μm	147.3 ± 61.4 μm	93.4 ± 61.3 μm	89.9 ± 55.1 μm

Figure S3

A) Neurite outgrowth analysis representative images of MAP2 (magenta) immunocytochemical characterization of neurons at DIV 7, 14, 21, and 28. B) Mean neurite length for each line at each time point with standard deviation. Scale bars: 50 µm.

Figure S4

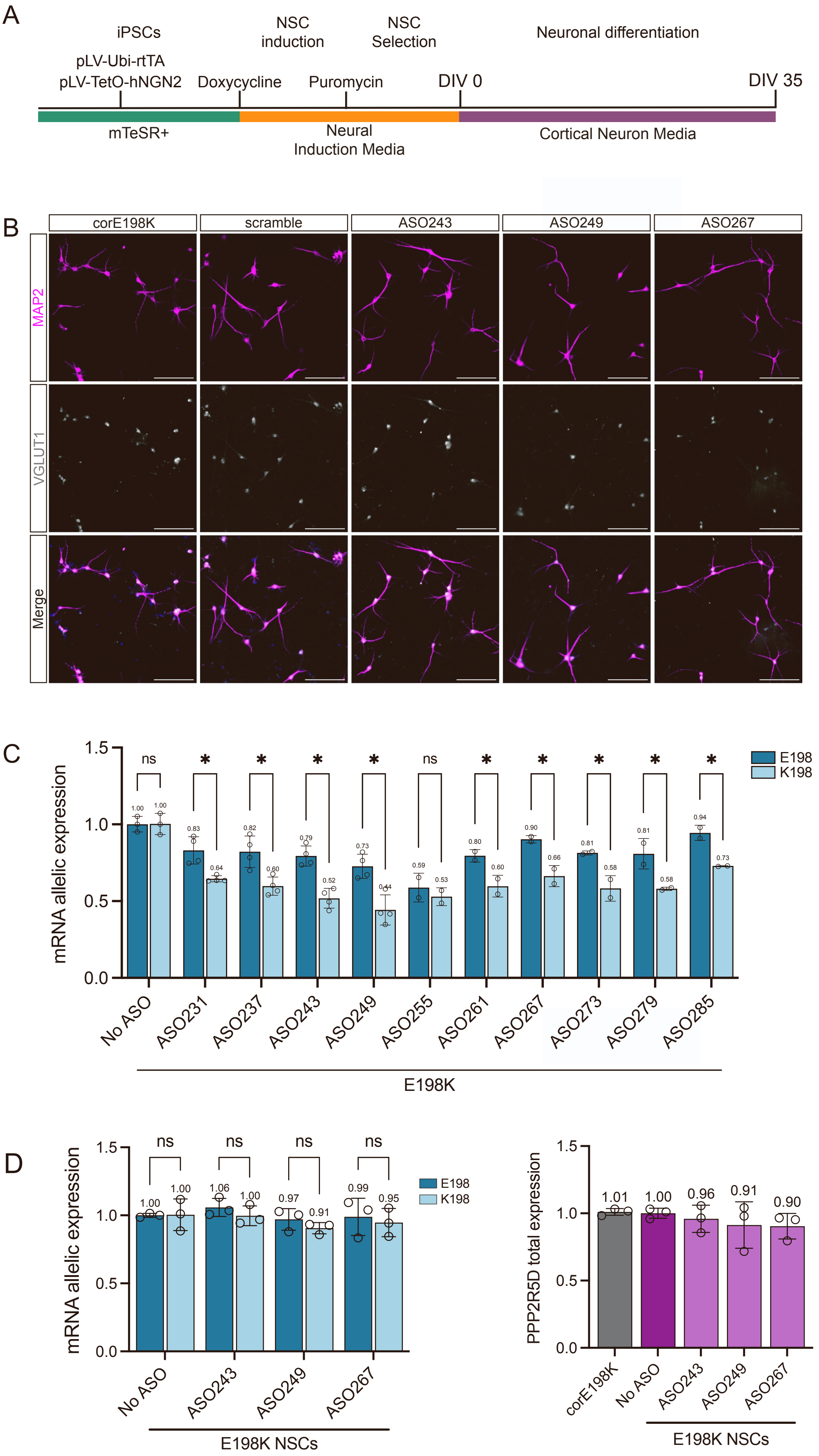
A
iPSCs
NSC induction
NSC Selection
Neuronal differentiation
pLV-Ubi-rtTA
pLV-TetO-hNGN2
Doxycycline
Puromycin
DIV 0
DIV 35
mTeSR+
Neural Induction Media
Cortical Neuron Media
B
corE198K
scramble
ASO243
ASO249
ASO267
MAP2
VGLUT1
Merge
C
mRNA allelic expression
E198
K198
No ASO
ASO231
ASO237
ASO243
ASO249
ASO255
ASO261
ASO267
ASO273
ASO279
ASO285
E198K
D
mRNA allelic expression
No ASO
ASO243
ASO249
ASO267
E198K NSCs
PPP2R5D total expression
corE198K
No ASO
ASO243
ASO249
ASO267
E198K NSCs

Figure S4

A) Schematic representation of the rapid iNGN2 neuronal differentiation protocol with time line and media supplements. B) Representative images of immunocytochemical characterization of MAP2 (magenta) and VGLUT1 (white) of iNGN2 neurons at DIV11. C) qRT-PCR quantification of allele-specific mRNA expression of E198K patient-derived iNGN2 neurons treated with ASOs for 3 days. D) qRT-PCR quantification of allele-specific mRNA expression and total *PPP2R5D* expression of E198K patient-derived NSCs treated with ASOs for 3 days. Data are represented as the mean $\pm$ SD. p-values ns: not significant, *: $p<0.05$.

Supplemental Tables

Table S1

List of all antibodies used in study.

Primary Antibodies				
Target	Host	Company	Catalog #	Dilution used
OCT4A	Rabbit	Cell Signaling Technologies	2840S	1:400
SOX2	Mouse	Cell Signaling Technologies	4900S	1:250
SSEA4	Mouse	Invitrogen	41-4000	
NESTIN	Mouse	Neuromics	MO22183	1:500
PAX6	Rabbit	Invitrogen	42-6600	1:250
KI67	Rabbit	Invitrogen	MA5-14520	1:500
MAP2	Mouse	Invitrogen	13-1500	1:500
PPP2R5D	Rabbit	Abcam	ab188323	1:300
VGLUT1	Guinea Pig	Synaptic Systems	135 304	1:1000
Secondary Antibodies				
Species Reactivity	Host	Company	Catalog #	Dilution used
Mouse, AF 488	Goat	Invitrogen	A32723	1:500
Mouse, AF 647	Goat	Invitrogen	A32728	1:500
Rabbit, AF 488	Goat	Invitrogen	A32731	1:500
Rabbit, AF 594	Goat	Invitrogen	A32740	1:500
Chicken, AF 488	Goat	Invitrogen	A32931	1:500
Chicken, AF 555	Goat	Invitrogen	A32932	1:500
Guinea Pig, AF 647	Goat	Invitrogen	A-21450	1:500

Table S2.

List of all primers used in study.

Application	Primer name	Sequence 5' --> 3'
qRT-PCR	qPPP2R5D-F	GAGGCCATTTACCCTGAGGC
	qPPP2R5D-R	TGTGGGATTCGATGAAGGTGG
	qGAPDH-F	TCAAGGCTGAGAACGGGAAG
	qGAPDH-R	CGCCCCACTTGATTTTGGAG
	qDSCAM-F	ATCAGACCCAGCGAACTCAG
	qDSCAM-R	CCAGCGGTAATCTGGCTCAG
	qNTN1-F	ACAACCCGCACAACCTGAC
	qNTN1-R	GGGACAGTGTGAGCGTGAC
	qRET-F	ACACGGCTGCATGAGACA
	qRET-R	GCCCTCACGAAGGGATGTG
	qSLITRK4-F	CCTGATTTCTTCGACAAATGCAG
	qSLITRK4-R	TCTCACAGTTGACATAGAGCACA
Sanger Sequencing	E198K-F	CTGCCACCTTCATCGAATCC
	E198K-R	CTTGGCTATGTTTGGCTGGA
	E420K-F	GCAAAGTGATGGAACCCCTC
	E420K-R	CGGGCAGCATTGTCACTTAT
	KO-F	ATTTTGCGGGGAAGGGAGTT
	KO-R	TGAAAACTGAGGGGTAGGGC
qRT-PCR probes	E198	5YAkYel/CCA+GA+G+G+AA+G+ATGA/3IABkFQ
	K198	56-FAM/CC+AGA+G+A+AA+G+ATGA/3IABkFQ
Amplicon Sequencing	E198K_wa-F	ACACTCTTTCCCTACACGACGCTCTTCCGATCTtgatgttgtcactgaggcca
	E198K_wa-R	GACTGGAGTTCAGACGTGTGCTCTTCCGATCTgtgatgctccgtctcgtaga
